# *Clostridioides difficile* minimal nutrient requirements for flagellar motility

**DOI:** 10.3389/fmicb.2023.1172707

**Published:** 2023-03-30

**Authors:** Julian Schwanbeck, Ines Oehmig, Uwe Groß, Wolfgang Bohne

**Affiliations:** ^1^Institute for Medical Microbiology and Virology, University Medical Center, Göttingen, Germany; ^2^Biotechnology Institute, University of Minnesota, Saint Paul, MN, United States

**Keywords:** *Clostridioides difficile*, nutrients, motility, flagella, minimal medium optimization

## Abstract

As many gastro-intestinal pathogens, the majority of *Clostridioides difficile* strains express flagella together with a complete chemotaxis system. The resulting swimming motility is likely contributing to the colonization success of this important pathogen. In contrast to the well investigated general energy metabolism of *C. difficile*, little is known about the metabolic requirements for maintaining the ion motive force across the membrane, which in turn powers the flagellar motor. We studied here systematically the effect of various amino acids and carbohydrates on the swimming velocity of *C. difficile* using video microscopy in conjunction with a software based quantification of the swimming speed. Removal of individual amino acids from the medium identified proline and cysteine as the most important amino acids that power swimming motility. Glycine, which is as proline one of the few amino acids that are reduced in Stickland reactions, was not critical for swimming motility. This suggests that the ion motive force that powers the flagellar motor, is critically depending on proline reduction. A maximal and stable swimming motility was achieved with only four compounds, including the amino acids proline, cysteine and isoleucine together with a single, but interchangeable carbohydrate source such as glucose, succinate, mannose, ribose, pyruvate, trehalose, or ethanolamine. We expect that the identified “minimal motility medium” will be useful in future investigations on the flagellar motility and chemotactic behavior in *C. difficile*, particularly for the unambiguous identification of chemoattractants.

## Introduction

The obligate anaerobic intestinal pathogen *Clostridioides* (*Clostridium*) *difficile* is currently the most prevalent cause of nosocomial diarrhea, infectious enteritis and pseudomembranous colitis in the Western world (Leffler and Lamont, 2015). As for many gastro-intestinal pathogens, flagellar based motility appears to be an important pathogenicity factor that influences colonization success (Stevenson et al., 2015). This was demonstrated on non-motile *C. difficile* mutants with defects in flagellar genes, which displayed reduced colonization efficiency in mice (Baban et al., 2013; Batah et al., 2017).

Bacterial flagellar motility is usually linked to a sensory system to achieve chemotaxis (Wuichet and Zhulin, 2010; Porter et al., 2011). All *C. difficile* genomes, with the exception of the non-motile clade 5, predict a complete chemotaxis operon with a single chemoreceptor (*mcp*) and further eight chemosensory genes, namely, the core components *cheA, cheY, cheR, cheB*, two variants of *cheW*, and two genes encoding the auxiliary proteins *cheC* and *cheD* (Dannheim et al., 2017). Functional genetic studies on these elements were not described yet and biochemical evidence for ligand binding to the chemoreceptor is also lacking.

Only a few studies addressed the chemotactic behavior in *C. difficile* so far. Engevik et al. (2021) quantified chemotaxis with the aid of fluorescently tagged bacteria in capillar assays and identified several monosaccharides, which are released after mucin digestion, as chemoattractants. N-Acetyl-D-neuraminic acid, also a component of mucin, was described before as a chemoattractant in a study that analyzed chemotaxis with the aid of video microscopy, although data were not processed automatically, but manually on a relatively small number of bacteria (Courson et al., 2019).

Surprisingly little knowledge exists on the general swimming behavior of *C. difficile* on the single cell level. The vast majority of studies that compared the swimming motility of *C. difficile* strains and mutants used easy to perform soft-agar based assays, which measure motility on the population level (Stevenson et al., 2015). We recently investigated single cell motility and swimming behavior of *C. difficile* with the aid of a bacterial tracking program (YSMR), which generates quantitative data on motility parameter such as speed, turning points and run lengths, simultaneously from several hundred bacteria (Schwanbeck et al., 2020). We could demonstrate that every motile *C. difficile* clade requires specific visco-elastic properties of the medium in order to display flagellar driven swimming motility (Schwanbeck et al., 2021). A motility pattern with long run phases only occurred when the matrix elasticity was increased by high molecular weight molecules, as for example, polyvinylpyrrolidones (PVP) or mucins. The motility behavior of *C. difficile* appears thus to be well adapted to the mucin-rich mucosal layer of the gastrointestinal tract (Schwanbeck et al., 2021).

As in PBS without metabolites no swimming activity was observed, even when high molecular weight molecules, such as PVP, are present, it can be assumed that *C*. *difficiles* swimming motility depends on the presence of nutrients in the medium, presumably in order to drive the flagellum (Schwanbeck et al., 2021). In the current study, we systematically investigated the effect of various amino acids and carbohydrates on the swimming velocity of *C. difficile*. This allowed us to speculate on the metabolic pathways involved in maintaining the ion motive force (IMF), which powers the flagellar motor. Furthermore, our studies defined a minimal set of nutrients that is sufficient to provide the energy for a swimming speed at maximum level. We expect that the identified “minimal motility medium” will be useful for future investigations on flagellar motility and chemotaxis in *C. difficile*.

## Materials and methods

For all experiments strain *C. difficile* 630 Δ*erm* was used (DSM 28645, CP016318.1) (Dannheim et al., 2017).

### Media and strain cultivation

Cultivation was always performed under anaerobic conditions using a COY anaerobic gas chamber (COY Laboratory Products, Grass Lake, MI, United States). The chamber was gas-flushed with 85% N_2_, 10% H_2_, and 5% CO_2_ and continuously surveilled for the presence of oxygen. *C. difficile* was grown at 37°C on Columbia agar with 5% sheep blood (COS, bioMérieux, Nürtingen, Germany). For experiments, single cultures were picked from plates and inoculated in 4 ml BHIS (37 g/l brain heart infusion broth supplemented with 5 g/l yeast extract, with 0.3 g/l cysteine added after autoclavation) shaking at 180 rpm over night.

### Sample preparation for motility experiments

The experimental setup, video acquisition and evaluation was similar as described before (Schwanbeck et al., 2021). From an overnight culture 4 ml BHIS were inoculated to an OD_600_ of 0.05 and grown while shaking at 180 rpm to an OD_600_ of 0.4–0.6. The exact OD_600_ was determined and an equivalent volume to 0.5 ml of OD_600_ 0.6 was centrifuged for 5 min at 1,500 × *g*. All centrifugation steps were performed at slow acceleration and slow brake ramps in order to preserve the flagella. The supernatant was discarded carefully without disturbing the pellet and the culture resuspended in 1 ml Dulbecco’s phosphate buffered saline (PBS; Merck, Darmstadt, Germany, order nr. D5652). For resuspension the reaction tubes were inverted and flicked in order to gently resuspend the pellet. The culture was centrifuged as before, then washed again in 1 ml PBS and centrifuged again. Subsequent steps were performed in the COY anaerobic gas chamber. After discarding the supernatant the pellet was resuspended in 0.5 ml PBS with 3.6% (w/v) polyvinylpyrrolidone (PVP K-90) (MW 360,000 g/mol, Carl Roth, Karlsruhe, Germany, order nr. CP15.1) and with a supplementation of amino acids and carbohydrates as described in the experiments. The components were used in the following concentrations: methionine (200 mg/L), cysteine (200 mg/L), isoleucine (300 mg/L), tryptophan (100 mg/L), proline (2,000 mg/L), leucine (400 mg/L), valine (300 mg/L), and 0.2 g/L of the indicated carbohydrate. No other concentrations were tested. Low molecular weight components, such as the added amino acids and carbohydrates do not affect matrix elasticity. From this mixture, 4 μl were applied to a microscopy slide, covered with a cover slip and sealed with nail polish. Subsequent video microscopy and single cell tracking were performed as previously described (Schwanbeck et al., 2021): 15 min after sealing, the slide samples were filmed for 3 min using a Nikon Eclipse TE2000-S microscope with a Nikon PlanFluor 10 × objective and a camera with an Aptina CMOS Sensor 18 MP 1/2.3 at 30 fps. Subsequently the video files were analyzed with YSMR v1.1 for median speed of the population.

### Statistical analysis

Statistical analysis was performed using the Tukey HSD method from the SciPy python package v1.10.1.

## Results

**FIGURE 1:**
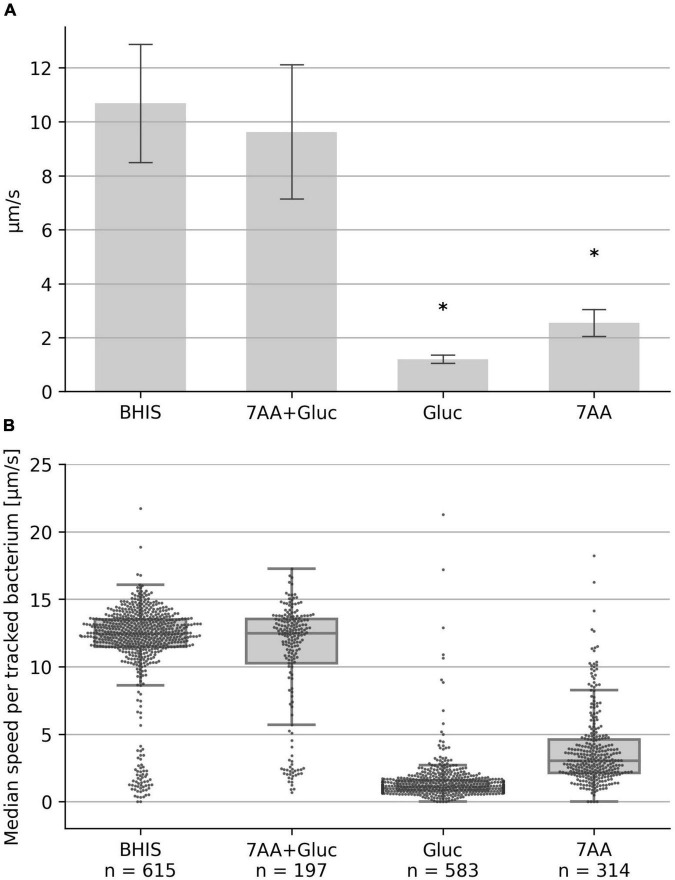
Swimming speed in defined medium. The median swimming speed (μm/s) of *Clostridioides difficile* cells was determined in (i) BHIS based medium (10% BHIS in PBS); (ii) PBS supplemented with the seven amino acids methionine (200 mg/L), cysteine (200 mg/L), isoleucine (300 mg/L), tryptophan (100 mg/L), proline (2,000 mg/L), leucine (400 mg/L), valine (300 mg/L), and 0.2 g/L glucose; (iii) PBS supplemented with the seven amino acids as before, but without glucose; (iv) PBS supplemented with 0.2 g/L glucose. **(A)** For each experiment, the median swimming speed of at least 100 bacteria was determined using the YSMR software. Indicated values are the mean from at least three independent experiments (biological replicates) ± standard deviation. Samples with significantly reduced values using the Tukey HSD method (*p* < 0.05) in comparison to the BHIS control were labeled with asterisks (*). Swimming speed was strongly reduced without glucose (bar 3) and without amino acids (bar 4). **(B)** One representative experiment. Each dot represents the median speed of one tracked bacterium, with the number of tracked bacteria per experiment given at the bottom (n). Additionally for each condition a box-plot is shown, indicating the interquartile range (IQR) (lower and upper end of the gray box), the median (middle line), and the lower/upper whisker limit as given by 1.5 times the IQR.

We recently demonstrated that *C. difficle* displays high motility in BHI medium supplemented with cysteine (BHIS), when viscoelasticity was increased by PVP addition (Schwanbeck et al., 2021). In the current study, we aimed to identify the essential nutrients in the medium that provide the energy for flagellar motility. BHI is a complex nutrient-rich, however, poorly defined medium in terms of exact composition of individual amino acids and carbohydrates and we thus searched the literature for a defined medium with a low number of nutrients that supports *C. difficile* growth. Neumann-Schaal et al. (2015) described a defined minimal medium that consists of seven amino acids, glucose, vitamins and salts. We tested a mixture of glucose and these seven amino acids, namely, methionine, cysteine, isoleucine, tryptophan, proline, leucine, valine in an YSMR-based motility assay and found that *C. difficile* motility, as measured by the median speed of the population, was identical to a BHIS control (Figure 1). However, the seven amino acids without glucose or glucose without amino acids resulted in strongly reduced motility, indicating that both components, amino acids plus a carbohydrate source, are required for motility (Figure 1). Representative videos are provided as Supplementary Files: Video 1 shows seven amino acids without glucose, Video 2 shows the control, and Video 3 shows glucose without amino acids.

**FIGURE 2:**
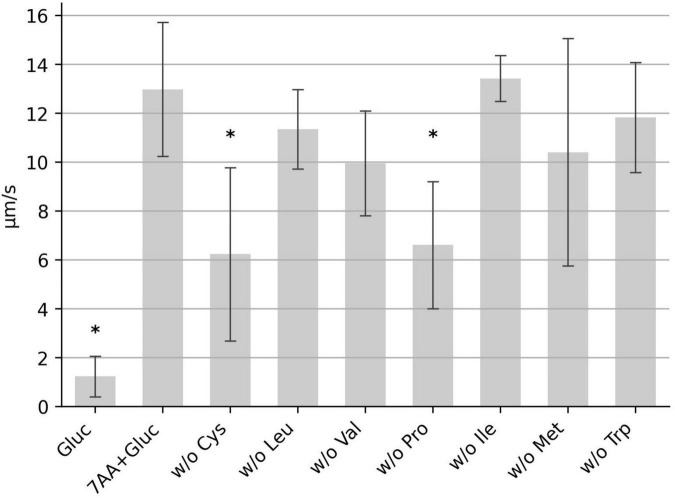
The median swimming speed (μm/s) of *C. difficile* cells was determined for different combinations of six amino acids. The control (7AA + Gluc) consists of PBS supplemented with the seven amino acids as described in Figure 1. The other samples include six out of these seven amino acids, without the indicated amino acid. All samples include 0.2 g/L glucose. For each experiment, the median swimming speed of at least 100 bacteria was determined using the YSMR software. Indicated values are the mean from at least three independent experiments (biological replicates) ± standard deviation. Samples with significantly reduced values using the Tukey HSD method (*p* < 0.05) in comparison to the control sample (7AA + Gluc) were labeled with asterisks (*). Swimming speed was significantly reduced without cysteine and without proline.

**FIGURE 3:**
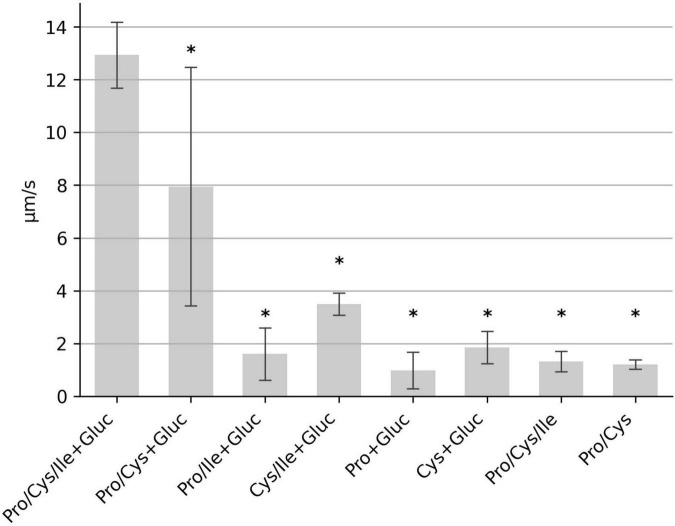
Identification of the “minimal motility medium.” The median swimming speed (μm/s) of *C. difficile* cells was determined for various combinations of proline (2,000 mg/L), cysteine (200 mg/L), isoleucine (300 mg/L), and glucose (0.2 g/L). For each experiment, the median swimming speed of at least 100 bacteria was determined using the YSMR software. Indicated values are the mean from at least three independent experiments (biological replicates) ± standard deviation. Samples with significantly reduced values using the Tukey HSD method (*p* < 0.05) in comparison to the sample containing proline, cysteine, isoleucine + glucose (PCI + Gluc) were labeled with asterisks (*). Swimming speed was strongly reduced in all samples, which lack either cysteine, or proline, or both.

We next determined which of the seven amino acids are most important for motility. We tested mixtures of six amino acids with one of the original amino acids lacking, respectively. Deprivation of proline and cysteine had the most severe effect on motility, while lack of methionine, isoleucine, tryptophan, leucine or valine only weakly effected *C. difficile* motility (Figure 2). In order to determine the minimal composition of amino acids required for maximum motility, we tested a combination of proline and cysteine with glucose. This combination resulted in a moderate average motility of 8 μm/s. However, the determined median speed values for the individual experiments (*n* = 10) showed a high degree of variability ranging from 2.5 to 13.3 μm/s (Figure 3). We found that addition of isoleucine resulted in a more stable motility of 12–13 μm/s, which is the maximal motility seen in samples with the seven amino acids plus glucose (Figure 2), as well as in previous experiments performed with BHIS (Schwanbeck et al., 2021). Thus, a combination of the three amino acids proline, cysteine and isoleucine is sufficient to obtain a maximum flagellar motility in *C. difficile* in the presence of glucose (Figure 3). This motility was stable for at least 1 h under continuous microscopic observation.

**FIGURE 4:**
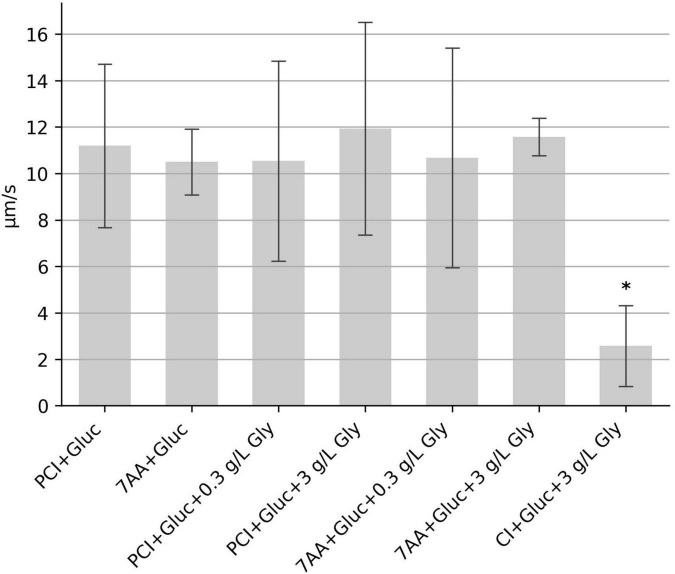
Influence of glycine on the swimming motility. The median swimming speed (μm/s) of *C. difficile* cells was determined (i) with additional glycine (bars 3–6), and (ii) by replacement of proline for glycine (bar 7). Concentration of glycine was as indicated either 0.3 g/l or 3 g/L. For each experiment, the median swimming speed of at least 100 bacteria was determined using the YSMR software. Indicated values are the mean from at least three independent experiments (biological replicates) ± standard deviation. Samples with significantly reduced values using the Tukey HSD method (*p* < 0.05) in comparison to the sample containing proline, cysteine, isoleucine + glucose (PCI + Gluc) were labeled with asterisks (*). Swimming speed was strongly reduced after replacement of proline for glycine.

*Clostridioides difficile* depends on the Stickland metabolism for energy production. In contrast to the oxidative branch of Stickland reactions, in which a variety of amino acids can be metabolized, only a few amino acids are used in the reductive pathway, among them proline and glycine. Glycine was not present in the initial mixture of seven amino acids. When these seven amino acids or samples consisting of proline, cysteine and isoleucine were supplemented with two different glycine concentrations, motility was almost unchanged. Only the sample with 3 g/L glycine added to the mixture of proline, cysteine, isoleucine and glucose (PCI + Gluc + 3 gL Gly) showed a marginal increase of 6.6% in comparison to the control sample without glycine (PCI + Gluc), which was statistically not significant (*p* = 0.83); (Figure 4). We also asked, whether proline can be replaced by glycine and tested a combination glycine, cysteine, isoleucine and glucose. This sample showed a strong reduction in the median speed (Figure 4), indicating that glycine is not able to replace proline to fuel motility.

**FIGURE 5:**
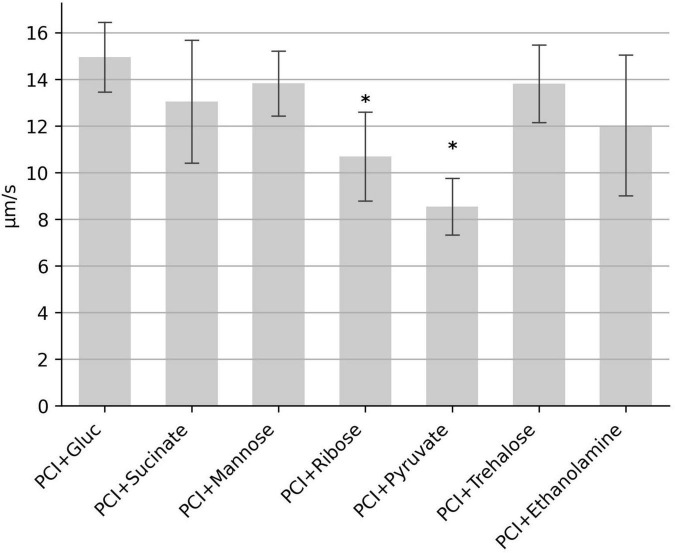
Influence of different carbohydrates on the swimming motility. The median swimming speed (μm/s) of *C. difficile* cells was determined in samples containing the three amino acids proline (2,000 mg/L), cysteine (200 mg/L), isoleucine (300 mg/L) (abbreviated as “PCI”) plus one of the indicated carbohydrates at (0.2 g/L). For each experiment, the median swimming speed of at least 100 bacteria was determined using the YSMR software. Indicated values are the mean from at least three independent experiments (biological replicates) ± standard deviation. Samples with significantly reduced values using the Tukey HSD method (*p* < 0.05) in comparison to the sample containing proline, cysteine, isoleucine + glucose (PCI + Gluc) were labeled with asterisks (*). Swimming speed was significantly reduced in samples with ribose (*p* < 0,006) and with pyruvate (*p* < 0.002).

Since samples without glucose display strongly reduced motility (Figure 1), we investigated whether glucose can be replaced by other carbohydrates. The minimal set of the three amino acids proline, cysteine and isoleucine were supplemented with either succinate, mannose, ribose, pyruvate, trehalose or ethanolamine. Motility was observed in all samples, however, median speed in samples with pyruvate and ribose were reduced to 57 and 71%, respectively (Figure 5). Motility in samples with succinate, mannose, trehalose and ethanolamine was similar to that of glucose. Together, these results indicate that various carbohydrate sources in combination with the three amino acids proline, cysteine and isoleucine can generate the metabolic energy that powers motility.

## Discussion

Flagellar motility in *C. difficile* depends on the presence of nutrients and we describe here the minimal set of components that is required to achieve the maximal swimming speed. This defined “minimal motility medium,” composed of proline, cysteine, isoleucine and glucose, should be useful in further studies on fllagellar motility and particularly on chemotaxis in *C. difficile*. Interpretation of chemotaxis assays is difficult when the candidate substance is metabolized and the resulting additional energy is markedly increasing the activity of the flagellar motor. In this case a general increase in swimming motility might occur, which could lead to similar results as a directed movement toward a chemoattractant in commonly used assays that test the behavior on a population basis such as capillary assays and media plates with a low agarose concentration. In capillary assays the number of bacteria is quantified that swim out of, or into a capillary. As this is a stochastic behavior, an increase in speed in the test conditions can change this frequency and thereby change the result of the assay without interaction with the chemotaxis system (Adler, 1973; Elgamoudi and Korolik, 2022). This problem should not occur in chemotaxis assays that use the described “minimal motility medium,” since the included four metabolites already support the maximum swimming speed and additional chemotactic candidate components can be tested unambiguously in this setting.

Bacterial flagellar motility is powered by an IMF across the membrane (Armitage and Berry, 2020). Several studies have shown a linear relationship between the IMF and the speed of the flagellar motor for Gram-negative as well as Gram-positive bacteria (for review see Biquet-Bisquert et al., 2021). Quantification of the swimming speed as performed in our study here is in this context an alternative readout system to monitor the IMF. In fact, the analysis of the metabolic requirements for flagellar motility in *C. difficile* can be regarded as a search for metabolites that are sufficient to build-up and maintain the IMF across the membrane.

The IMF in bacteria can consist of a proton and/or a Na + gradient. Stator proteins of the flagellar motor are specific either for a proton motive force or for a sodium motive force (Chang et al., 2021; Guo and Liu, 2022). They translocate ions through a channel to the inner membrane site and convert the electrochemical gradient into torque, which ultimately leads to rotation of the rotor unit (Biquet-Bisquert et al., 2021). Activity of MotAB-type stators is coupled to H^+^ transport, while MotPS and PomAB-type stators are coupled to Na^+^ transport (Biquet-Bisquert et al., 2021). In most bacteria a single type of stator is expressed, however, some species express H^+^-dependent and Na^+^-dependent stators simultaneously (Thormann and Paulick, 2010). We inspected the genome of *C. difficile* 630Δ*erm* (GenBank accession number CP016318.1) for the presence of genes encoding *motA/B*, *motP/S*, and *pomA/B*, but could identify only a single *motA* gene (locus_tag: CDIF630erm_00380) and a single *motB* gene (locus_tag: CDIF630erm_00381). This suggests that the flagellar motor in *C. difficile* appears to be exclusively driven by a proton motive force.

In contrast to the general energy metabolism, a surprisingly small number of metabolites is sufficient to power the flagellar motor, namely, the amino acids proline and cysteine together with a single, but interchangeable carbohydrate. The importance of proline and cysteine for maintenance of flagellar motility was further stressed in experiments with minimal growth medium consisting of seven amino acids and glucose. When either proline or cysteine were depleted, a strong reduction in the swimming speed was observed.

The particular importance of proline for motility comes with little surprise, since this amino acid is the preferred substrate for the reductive Stickland pathway (Neumann-Schaal et al., 2019). In Stickland reactions the reduction of one amino acid is coupled with the oxidation of a second subset of amino acids. Our experiments suggest that cysteine is the preferred amino acid for the oxidative branch to maintain the IMF. This is unexpected, since cysteine is not among the amino acids that were previously described to be the most efficient electron donors for the oxidative branch (Neumann-Schaal et al., 2019; Marshall et al., 2023). The most stable and highest motility was observed when a third amino acid, isoleucine, was added to the motility medium. This suggests that in addition to cysteine, isoleucine might also be contributing as an electron donor in the oxidative branch. When proline and cysteine were the sole amino acids in the motility medium, the individual experiments showed a high degree of variability in bacterial motility. This might be due to slight variations in intracellular amino acid concentrations at the time when bacteria were harvested.

The crucial enzyme responsible for the generation of the IMF in *C. difficile* is the membrane spanning Rnf complex, which uses reduced ferredoxins as substrates and couples the electron transfer from ferredoxin to NAD+ with the generation of an ion gradient across the membrane (Biegel and Muller, 2010; Chowdhury et al., 2016). The reduced ferredoxins themselves are derived from Stickland reactions. The oxidative Stickland branch reduces ferredoxin by the activity of ferredoxin-dependent oxidoreductases, while in the reductive Stickland pathway, the NADH-dependent reduction of substrates are connected to ferredoxin reduction *via* electron bifurcation (Bertsch et al., 2013).

It was demonstrated for *Clostridium sporogenes* that proline reduction is directly linked to the generation of a proton motive force (Lovitt et al., 1986). To our knowledge, experimental evidence for this coupling was not published for *C. difficile* yet, although it was proposed that the selenoenzyme D-proline reductase directly interacts with the Rnf complex (Berges et al., 2018). In contrast, a direct coupling to the Rnf complex was not described for the reduction of glycine, although this amino acid, as proline, is an efficient electron acceptor in Stickland reactions; (Jackson et al., 2006; Kim et al., 2006; Marshall et al., 2023). Our observation that glycine could not replace proline in assays with a minimal number of additional compounds (cysteine, isoleucine and glucose) suggests a fundamental difference in the manner of how these two amino acids contribute to the IMF. A plausible explanation is that the D-proline reductase is indeed coupled to the activity of the Rnf complex and is thus of particular importance for IMF maintenance.

Proline metabolism was also shown to be essential for colonization in experiments in mice. A *C. difficile* mutant with a defect in proline metabolism was shown to be unable to infect germ-free mice (Battaglioli et al., 2018). It was also demonstrated that intestinal proline concentration is low with an intact microbiota, but strongly increases after dysbiosis. In fact, proline was shown to be the amino acid with the greatest difference in a comparison between healthy and dysbiotic mice (Battaglioli et al., 2018). Human donor fecal samples from patients with dysbiosis also showed increased proline levels (Battaglioli et al., 2018). Together, these *in vivo* data indicate that increased proline levels are strongly associated with *C. difficile* colonization success. Our observation that flagellar motility is proline dependent let to the hypothesis that flagellar motility during the course of infection might be restricted to proline-rich microenvironments, in which the motile bacteria can colonize and spread in a short period of time.

## Data availability statement

The raw data supporting the conclusions of this article will be made available by the authors, without undue reservation.

## Author contributions

JS, WB, and UG had the initial idea. JS and IO performed the experiments. WB wrote the manuscript. All authors read and approved the final text.
